# In vitro activity of a G-quadruplex-stabilizing small molecule that synergizes with Navitoclax to induce cytotoxicity in acute myeloid leukemia cells

**DOI:** 10.1186/s12885-019-6464-9

**Published:** 2019-12-27

**Authors:** Justin J. Montoya, Megan A. Turnidge, Daniel H. Wai, Apurvi R. Patel, David W. Lee, Vijay Gokhale, Laurence H. Hurley, Robert J. Arceci, Cynthia Wetmore, David O. Azorsa

**Affiliations:** 1 The Institute of Molecular Medicine at Phoenix Children’s Hospital, 475 N 5th Street, Phoenix, AZ 85004 USA; 2 Department of Child Health, The University of Arizona College of Medicine-Phoenix, 475 N 5th Street, Phoenix, AZ 85004 USA; 3 School of Life Sciences, Arizona State University, Tempe, AZ USA; 4 Present Address: Department of Molecular & Medical Genetics, Oregon Health and Science University, Portland, OR 97239 USA; 5 Center for Cancer and Blood Disorders, Phoenix Children’s Hospital, Phoenix, AZ USA; 6 University of Arizona College of Pharmacy, Tucson, AZ USA; 7 Present Address: Systems Oncology, Scottsdale, AZ 85255 USA

**Keywords:** MYC, G-quadruplex, AML, Bcl-2, Bcl-X_L_, Navitoclax

## Abstract

**Background:**

Acute Myeloid Leukemia (AML) is a malignancy of myeloid precursor cells that arise from genomic alterations in the expression of key growth regulatory genes causing cells to assume an undifferentiated state and continue to proliferate. Recent efforts have focused on developing therapies that target specific protein products of aberrantly expressed genes. However, many of the identified proteins are difficult to target and thought to be “undrugable” because of structural challenges, protein overexpression, or mutations that confer resistance to therapy. A novel technology that circumvents some of these issues is the use of small molecules that stabilize secondary DNA structures present in the promoters of many potential oncogenes and modulate their transcription.

**Methods:**

This study characterizes the in vitro activity of the G-quadruplex-stabilizing small molecule GQC-05 in AML cells. The effect of GQC-05 on three AML cell lines was analyzed using viability and apoptosis assays. GQC-05 has been shown to down-regulate MYC through G-quadruplex stabilization in Burkitt’s lymphoma cell lines. MYC expression was evaluated through qPCR and immunoblotting in the three AML cell lines following the treatment of GQC-05. In order to identify other therapeutic agents that potentiate the activity of GQC-05, combination drug screening was performed. The drug combinations were validated using in vitro cytotoxicity assays and compared to other commonly used chemotherapeutic agents.

**Results:**

GQC-05 treatment of KG-1a, CMK and TF-1 cells decreased cell viability and resulted in increased DNA damage and apoptosis. Additionally, treatment of KG-1a, CMK and TF-1 with GQC-05 resulted in decreased expression of MYC mRNA and protein, with a more pronounced effect in KG-1a cells. Combination drug screening identified the Bcl-2/Bcl-X_L_ inhibitor Navitoclax as a compound that potentiated GQC-05 activity. Co-treatment with GQC-05 and Navitoclax showed a synergistic decrease in cell viability of AML cells as determined by Chou-Talalay analysis, and induced more DNA damage, apoptosis, and rapid cytotoxicity. The cytotoxicity induced by GQC-05 and Navitoclax was more potent than that of Navitoclax combined with either cytarabine or doxorubicin.

**Conclusion:**

These results suggest that the G-quadruplex stabilizing small molecule GQC-05 induces down regulated MYC expression and DNA damage in AML cells. Treatment with both GQC-05 with a Bcl-2/Bcl-X_L_ inhibitor Navitoclax results in increased cytotoxic activity, which is more pronounced than Navitoclax or GQC-05 alone, and more significant than Navitoclax in combination with cytarabine and doxorubicin that are currently being used clinically.

## Background

Acute Myeloid Leukemia (AML) is a malignancy of poorly differentiated myeloid precursor cells that proliferate robustly, leading to abnormal hematopoiesis and eventually to bone marrow failure [1]. Genomic analysis has demonstrated that AML is composed of a heterogeneous population of malignant cells with numerous genetic complexities [2, 3]. These genetic aberrations often lead to dysregulation of proto-oncogenes such as CREB, MYC, EVi-1, MN-1 [4, 5], resulting in difficult to treat tumor cells and poor clinical outcome [6].

G-quadruplexes (G4) are secondary four-stranded helical structures of DNA comprised of guanine-rich nucleic acids that are enriched in the promoter regions of many genes. G4 are involved in many cellular processes including DNA replication, gene expression, telomere maintenance and apoptosis [7, 8]. Many genomic sequences contain areas of guanine rich regions that have the ability to self-associate to form G4, which are more thermodynamically stable than double stranded DNA [8]. It has been estimated that nearly 50% of human genes contain G4 s near their promotor regions, and are more frequently found near the promoters of oncogenes or regulatory genes than near housekeeping genes [9]. Since a dynamic equilibrium occurs between the promoter’s transcriptionally active single stranded, double stranded, and G-quadruplex forms, there exists the opportunity to repress gene transcription by stabilizing the G-quadruplex structure. Stabilization of G-quadruplex structures have been described previously in vitro using the porphyrin molecule TMPyP4 [10] and the ellipticine analog GQC-05 [11]. One of the first reports that G4 s near promotor regions can effect gene expression arose from studies of the c-MYC oncogene, demonstrating that mutation of the endogenous G4 sequence or addition of a G4-stabilizing ligand altered transcription in vivo [10].

MYC regulates many hallmarks of cancer such as cell growth, proliferation, cell-cycle, apoptosis, and cell differentiation, making it a potent oncogene when dysregulated [12] and an attractive, but as yet, “undruggable” therapeutic target [13]. The compound GQC-05 was shown to stabilize the G-quadruplex in the *MYC* promoter – among other growth regulatory genes - and also repress its transcription in Burkitt’s lymphoma [11].

In this present study, we sought to determine the effects GQC-05 on the expression of MYC and other genes, and to characterize the cellular consequences of AML cells exposed to GQC-05. We found a varied cytotoxic activity of GQC-05 in AML cells and we sought to characterize the mechanism of cell death induced by GQC-05. Furthermore, we completed a drug screen to identify potentiators of GQC-05 activity and demonstrated a novel synergy when GQC-05 was combined with the Bcl-2/Bcl-X_L_ inhibitor Navitoclax. These studies also demonstrate that GQC-05 can inhibit MYC expression as previously seen in Burkitt’s lymphoma [12]. GQC-05 also induces DNA damage response and induced cytotoxic activity that was increased by the addition of Navitoclax, thereby increasing its potential as therapeutic anti-cancer agent.

## Methods

### Cell culture

All cell lines were authenticated using Short Tandem Repeat (STR) analysis by the University of Arizona genomics core. The CMY [14], CMK [15], and CMS [16] cell lines were a generous gift from Dr. Jeffrey W. Taub, Wayne State University. The KG-1a cell line was grown in IMDM media (Corning) supplemented with 20% Fetal Bovine Serum (FBS; Atlas Biologicals), 1% L-Glutamine (Caisson Labs), and 1% penicillin/streptomycin (Gibco). The UT-7epo cells were grown in similar IMDM media that was supplemented with 1 U/mL recombinant erythropoietin (rhEPO; R&D Systems). The Molm-13, Kasumi-1, CMY, NB4, TF-1, M-07e, CMK, HEL, THP-1, U937, AML-193, and CMS cells were grown in RPMI 1640 (Corning) with 10% FBS and 1% penicillin/streptomycin and L-glutamine. The RPMI growth media for TF-1 and M-07e was supplemented with 10 ng/mL granulocyte macrophage colony-stimulating factor (GMCSF; R&D systems), and the media for AML-193 contained 2 ng/mL GMCSF as well as 5 μg/mL Insulin Transferrin Selenium A (ITS; Gibco). PBMCs were isolated from whole blood by density centrifugation using Ficoll (GE Life Sciences) and grown in RPMI (10% FBS) supplemented with 10 ng/mL IL-2 (R&D Systems). All cells were grown at 37 °C with 5% CO_2_. For 6 well plate assays, cells were plated at 1,500,000 cells/well (KG-1a and TF-1) or 1,000,000 cells/well (CMK). Cells were allowed to grow overnight before treatment.

### Antibodies, primers, and compounds

Primary antibodies for MYC (Rabbit mAb #5605), Bcl-2 (Rabbit mAb #2870, Mouse mAb #15071), phospho-histone H2A.X (Ser139) (Rabbit Ab #2577) and PARP (Rabbit mAb #9532) were purchased from Cell Signaling Technology. The GAPDH (Mouse mAb sc-166,545) primary antibody was purchased from Santa Cruz Biotechnology. Secondary Rabbit and Mouse antibodies used for chemi-luminescence were obtained from Jackson Immunoresearch. Secondary antibodies IRDye® 800CW GAR and IRDye® 680RD GAM used for near infrared western blot detection were obtained from LI-COR Biosciences.

Gene specific qPCR primers for MYC (Forward: 5′-GCCCACCACCAGCAGCGACTC-3′, Reverse: 5′-GCACCTCTTGAGGACCAGTGG-3′), YWHAZ (Fwd: 5′-AGAGAAAGCCTGCTCTCTTGC-3′, Rvs: 5′-AGGAGTGGGTGTCGCTGTTG-3′) were obtained from Life Technologies.

The G-quadruplex stabilizing compound GQC-05 was synthesized as previously described [11] at the School of Pharmacy, University of Arizona. The small molecules Navitoclax, (+)-JQ1, doxorubicin and cytarabine were obtained from Selleckchem (Houston, TX).

### Drug dose response viability assays

To determine the cytotoxic effects of compounds, AML cells (2000 cells/well) were plated into 384-well plates. At 24 h, cells were treated with various drug doses in quadruplicate wells for 72 h. Drug treatments consisted of 1:2 or 1:3 serial dilutions. After 72 h of treatment, Cell Titer-Glo® (Promega) was added to the cells, and luminescence was read using the PerkinElmer EnVision Multilabel Plate Reader. Samples were normalized to untreated controls and dose response curves and IC50 values were determined using GraphPad Prism. Drug synergy was determined using CompuSyn software (ComboSyn, Inc) for Chou-Talalay analysis [17].

### Western blots

**Fig. 1:**
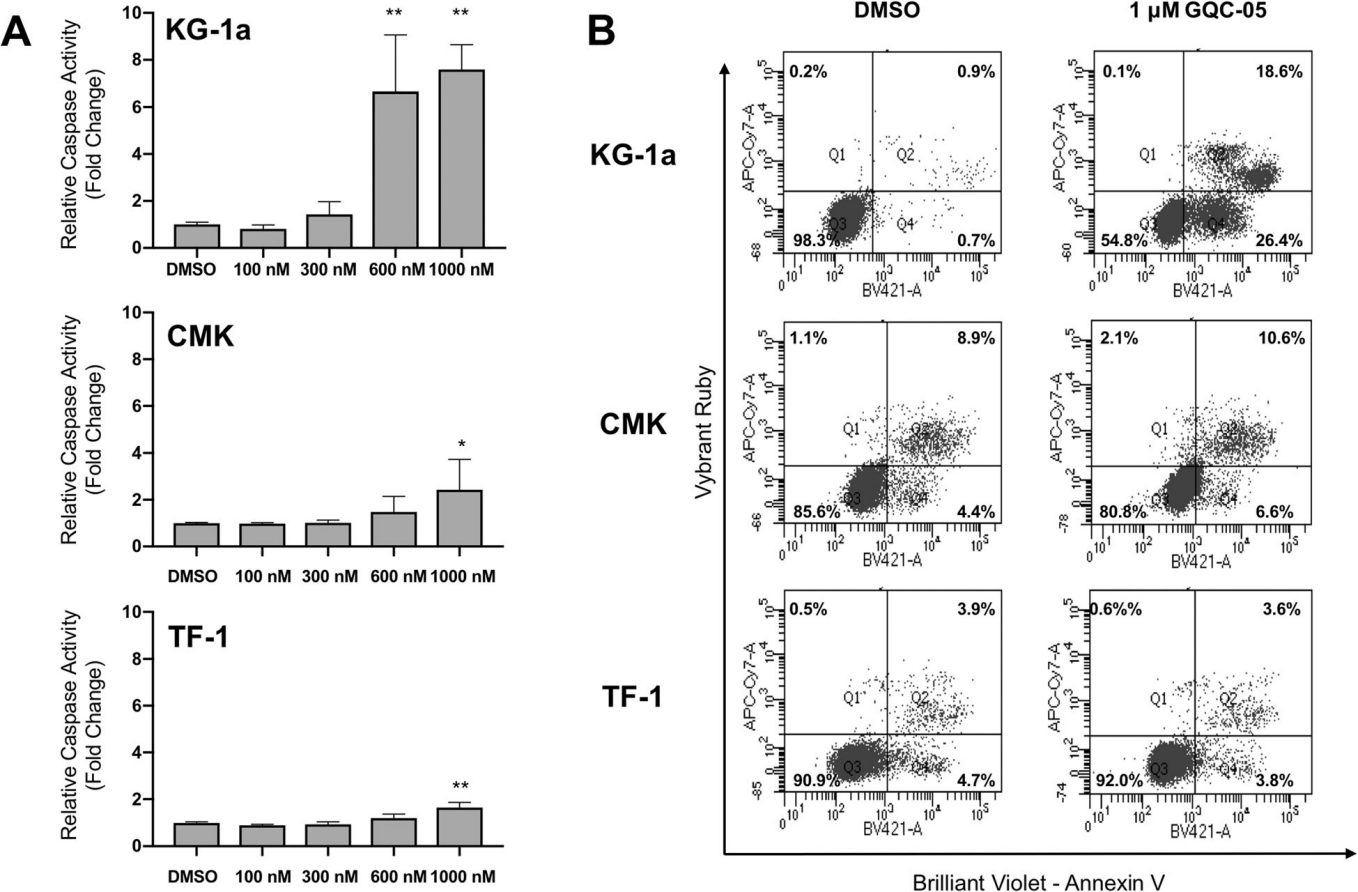
GQC-05 induces apoptosis and DNA damage in KG1a cells but not CMK or TF-1 cells. The AML cell lines KG-1a, CMK and TF-1 were treated with vehicle (DMSO), 100 nM, 300 nM, 600 nM, or 1000 nM GQC-05 for 24 h. **a** Treated cells were assayed for Caspase 3/7 activity, normalized to DMSO treated cells and shown as mean fold change +/− S.D. * P < 0.002,** *P < 0.0002* for quadruplicate samples of three biological replicates. **b** Apoptosis analysis using flow cytometry for Annexin V binding and nuclear (Vybrant Ruby) staining on AML cells treated with vehicle (DMSO) or 1000 nM GQC-05

Cells were plated at 2000,000 cells/well in 6 well plates and allowed to grow for 6 or 24 h after treatment. Treated cells were washed in PBS and lysed using RIPA lysis buffer (Thermo Fisher) supplemented with 1x Halt Protease & Phosphatase inhibitor cocktail (Thermo Fisher). Gel electrophoresis was performed on 15 or 10-well 1.5 mm NuPAGE 4–12% Bis-Tris gels (Thermo Fisher). Samples were then transferred to Invitrolon PVDF transfer membranes (Invitrogen) and blocked overnight at 4 °C or 1 h at room temperature in blocking buffer consisting of 1x TBS (Quality Biological) with 0.1% Tween® 20 (Thermo Fisher) and 5% dehydrated milk. Primary antibodies were used at concentrations of 1:1000 except the GAPDH control, which was 1:5000. The secondary antibody concentration used was at a 1:20,000 dilution. Blots were imaged after treatment with SuperSignal West Pico Chemiluminescent substrate (Thermo Fisher) using the ChemiDoc™ Imaging System (Bio-Rad). Bands were quantified using the Image Lab™ software (Bio-Rad). Figure 1b samples were prepared as described above and imaged using near infrared western blot detection. Blots were prepared as described above and probed with near infrared secondary antibodies IRDye® 800CW GAR and IRDye® 680RD GAM (LI-COR). Blots were imaged using LI-COR Odyssey FC and quantified using Empiria Studio™ Software (LI-COR).

### qRT-PCR

Cells were plated at a concentration of 2000,000 cells/well in 6 well plates and treated for 6 h. Total RNA was isolated from treated cells using the Qiagen RNeasy® Plus Mini Kit as per manufacturer instructions. RNA samples (1 μg) were reverse transcribed using the iScript Select cDNA synthesis kit (Bio-Rad). qRT-PCR reactions were prepared in triplicate using iQ SYBR GreenSupermix (Bio-Rad) and were performed using a CFX96 Real-Time qPCR Detection System (Bio-Rad). Analysis of fold change gene expression was performed on Excel (Microsoft) spreadsheets using the Comparative C_T_ method [15, 18].

### Drug library screening

KG-1a cells were plated into three 384-well plates at a concentration of 2000 cells per well using MultiFlo FX dispenser (BioTek). At 24 h, cells were treated with 53 compounds using five doses ranging from 1 nM to 10 μM in 10-fold increments. Compounds were added to plates from a drug screening master plate dispensed with the Acoustic Transfer System (ATS) (Biosero). Immediately following drug screen compound addition, DMSO control or GQC-05 was added to give an assay concentration of 0 nM (DMSO), 100 nM or 300 nM using the ATS. After 72 h of treatment, Cell Titer-Glo® (Promega) was added to the cells and luminescence was read using the EnVision Multilabel Plate Reader (PerkinElmer). Samples were normalized to DMSO controls. Dose response curves and Area Under the Curve (AUC) values were determined using GraphPad Prism. Combination effects were analyzed using AUC of dose response curves and compared to DMSO controls.

### Combination drug treatment

Cells were plated in 384-well plates at a concentration of 2000 cells per well using a 12-channel automated pipette (Rainin). At 24 h, drug is added in quadruplicated wells. AML cells were treated with a matrix of concentrations of GQC-05 ranging from 20 to 600 nM and Navitoclax ranging from 2 nM – 2 μM for 72 h. After 72 h of treatment, cell viability was assessed using Cell Titer-Glo® (Promega) and luminescence was read using the EnVision Multilabel Plate Reader (PerkinElmer). Samples were normalized to no treatment controls. Dose response curves and IC50 values were determined using GraphPad Prism.

### Luminescence-based caspase 3/7 activity analysis

Cells were plated in 6-well plates as described above. Aliquots (4 × 25 μl) were taken for apoptosis assays and transferred in quadruplicate to 384 well plates. Caspase-Glo® 3/7 (Promega) was used for the apoptosis assay to determine the activity of caspases 3 and 7, two enzymes used by cells during apoptosis. Caspase-Glo® 3/7 contains a substrate that, when cleaved by caspase 3 or 7, produces ATP. A thermostable luciferase ATPase also present in the solution emits luminescence proportional to the amount of ATP in the sample. Luminescence was measured using the EnVision Multilabel Plate Reader (PerkinElmer).

### Flow cytometry

Cells were plated and treated in 6 well plates as described above. After 24 h of treatment, 500,000 cell aliquots of each sample were taken, including 4 aliquots of the 10 μM Etoposide positive control and 1 aliquot of each other treatment. Cells were washed twice with cell staining buffer (1% FBS in PBS), and then resuspended in 95 μL 1X Annexin V binding buffer (ThermoFisher) containing Vybrant® Cell Cycle Ruby dye (ThermoFisher) and Brilliant Violet-421™-Annexin V (BioLegend) as per manufacture’s instruction. For combination studies, cells were stained with 1X Annexin V binding buffer (Invitrogen) with 5 μM Sytox® Red dead cell stain (Life Technologies) and Annexin V Alexa Flour™488 conjugate (1:20 dilution) (ThermoFisher). Instrument controls included Sytox® only, Annexin V only, or unstained sample. Samples were then incubated in the dark at room temperature for 15 min and diluted 1:10 in PBS prior to analysis. Flow cytometric analysis was performed at the University of Arizona Translational Flow Cytometry Laboratory on a FACSCanto flow cytometry system (BD Biosciences) or Guava® easyCyte™ flow cytometer (Millipore). Acquired data was analyzed using FlowJo analysis software.

### RealTime-Glo™ Annexin V apoptosis necrosis assay (Promega)

Cells were added to a 384 well plate at a concentration of 2500 cells per well, as per manufacturer recommendation and incubated for 18 h at 37 °C with 5% CO_2_. Two hours prior to drug addition, 2X RTG detection reagent was added to all wells. At 24 h, drug was added in quadruplicate wells. Cells were treated with DMSO, GQC-05, Navitoclax, or GQC-05 in combination with Navitoclax. Fluorescence and luminescence were read using the EnVision Multilabel Plate Reader (PerkinElmer) at 1-h increments up to 6 h and another read at 24 h. Samples were normalized to untreated controls. Dose response curves were generated using GraphPad Prism.

## Results

### GQC-05 induces apoptosis in AML cells

**Fig. 2:**
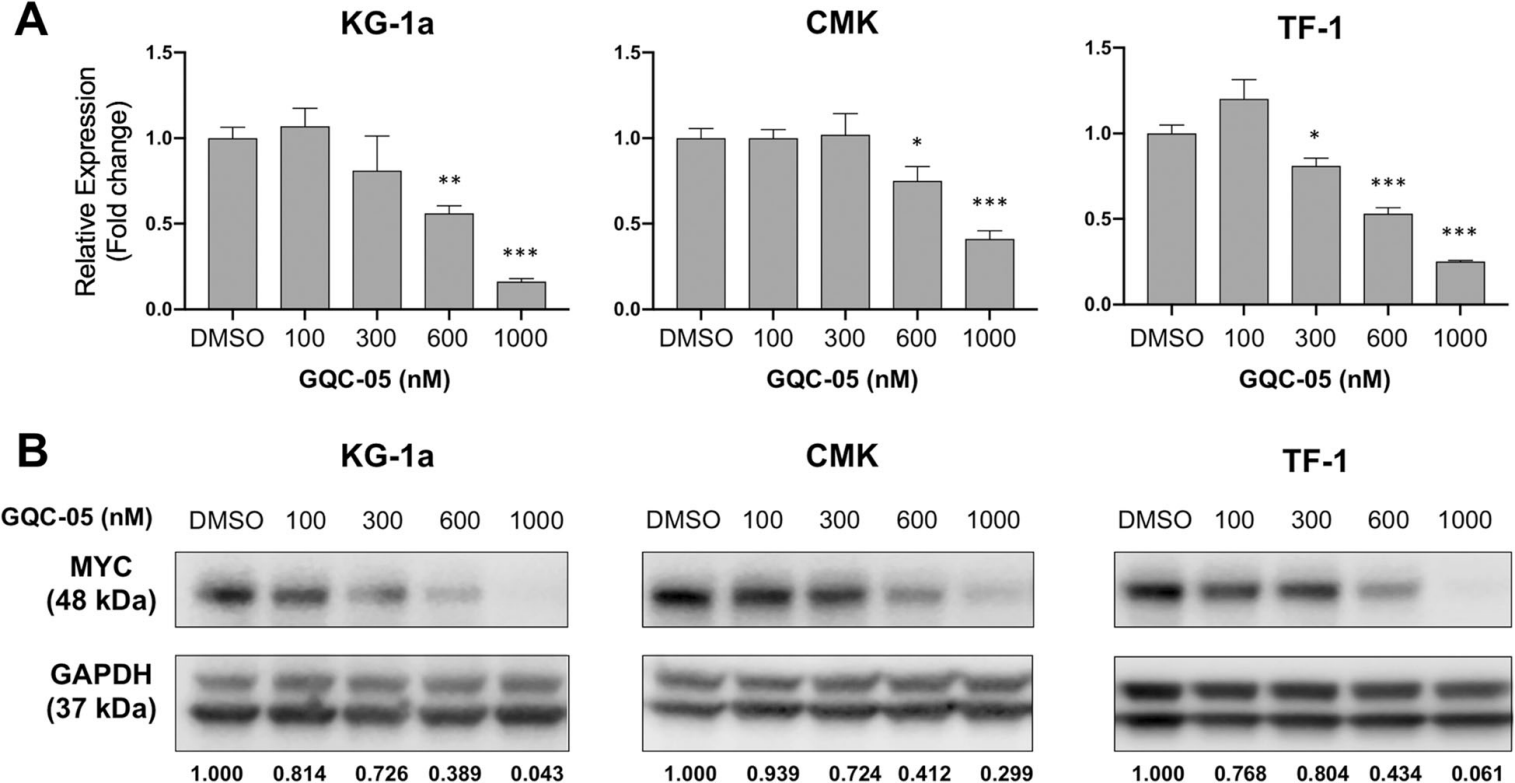
The G-quadruplex stabilizer GQC-05 induces rapid downregulation of MYC in AML cells. The AML cell lines KG-1a, CMK and TF-1 were treated with vehicle (DMSO), 100 nM, 300 nM 600 nM or 1 μM GQC-05 for 6 h. **a** qRT-PCR analysis for *MYC* expression in vehicle (DMSO) or GQC-05 treated AML cells. Data normalized to YWHAZ as +/− mean and is representative of three biological replicates. * *P* < 0.01, ** *P* < 0.002, *** *P* < 0.0002 for triplicate samples. **b** Western immunoblotting analysis of MYC expression in whole cell lysates from DMSO or GQC-05 treated AML cells. Band intensity ratios were calculated foe MYC relative to the loading control GAPDH

AML cell lines KG-1a, CMK, and TF-1 were treated with GQC-05 for 24 h and analyzed for induction of caspase 3/7 as an indication of apoptosis. Caspase 3/7 activity was induced in KG-1a cells treated with 600 nM and 1 μM GQC-05 and in CMK and TF-1 cells treated with 1 μM GQC-05 (Fig. 2a). Increased annexin V staining in was also induced by treatment with GQC-05 with the greatest increase in KG-1a cells (Fig. 2b), suggesting that KG-1a cells are more sensitive to the effects of GQC-05.

### GQC-05 exposure decreased MYC mRNA in AML cells

We evaluated the activity of GQC-05 on cell viability of a panel of AML cells and determined the relative EC50 values, noting the variability in sensitivity to GQC-05 as well as the sensitivity to cytarabine, as a positive control (Table 1). Cellular response to GQC-05 varied among lines with EC50 values ranging from 4 nM to 400 nM. We selected lines KG-1a, CMK and TF-1 for further studies due to their high expression of MYC and varied response to GQC-05. We found that GQC-05 treatment decreased expression of MYC mRNA in the AML cell lines, as well as decrease in MYC protein at 6 h. (Fig. 1).

**Table 1:** EC50 Values of AML Cells Treated with GQC-05 or Cytarabine

Cell Line	GQC-05 (nM)	Cytarabine (nM)
Molm-13	4 ± 0.9	4 ± 0.9
M-07e	70 ± 29	24 ± 4.4
U937	213 ± 109	27 ± 2.9
HEL	203 ± 90	34 ± 4.3
Kasumi-1	44 ± 14	60 ± 57
NB4	113 ± 17	65 ± 34.8
CMK	157 ± 26	75 ± 5
TF-1	128 ± 40	103 ± 26
KG-1a	89 ± 30	157 ± 31
CMS	220 ± 48	247 ± 162
AML-193	321 ± 52	414 ± 30
CMY	104 ± 14	1588 ± 353
THP-1	194 ± 76	4224 ± 564
UT-7epo	76 ± 32	6821 ± 3880

### Identification of Navitoclax as a potential cytotoxic agent in combination with GQC-05

**Fig. 3:**
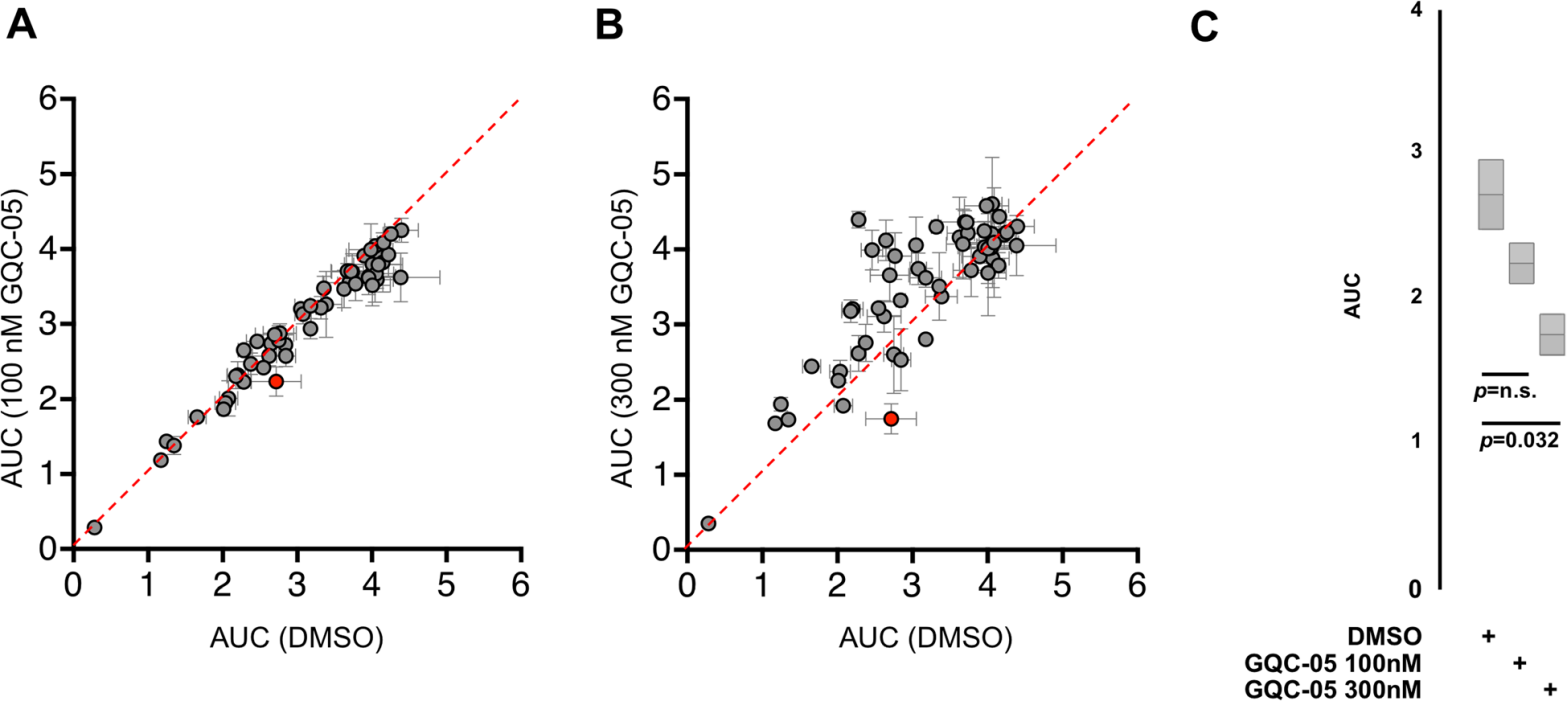
Combination drug screen identifies Navitoclax as a potential sensitizer to GQC-05. The AML cell line KG-1a was treated with a library of 53 cancer drugs or inhibitors at doses ranging from 1 nM – 10 μM and either vehicle (DMSO), 100 nM GQC-05 or 300 nM GQC-05. Cells were assayed for cell number after 72 h of treatment and normalized to untreated cells. Dose response curves were determined for each drug library compound and Area Under the Curve (AUC) was calculated from two biological replicate screens and plotted for (**a**) DMSO vs 100 nM GQC-05 or (**b**) DMSO vs 300 nM GQC-05. **c** AUC values for cells treated with the combination of Navitoclax and either DMSO, 100 nM GQC-05 or 300 nM GQC-05

We next performed a small molecule combination screen using a panel of 54 cancer drugs and inhibitors to identify potential combination partners that potentiate GQC-05 activity in KG-1a cells. (Additional file 1: Table S1). Cell viability was assessed and activity of the small molecules was determined by calculating the Area Under the Curve (AUC) for each small molecule in combination with either a high or low dose of GQC-05 and compared to vehicle treated (Fig. 3a–b, Additional file 1: Table S1). In combination with 300 nM GQC-05, the AUC of the Bcl-2/Bcl-X_L_ inhibitor Navitoclax was significantly lower compared to vehicle treated (Fig. 3c) indicating potentiation of GQC-05 activity.

### GQC-05 acts synergistically with Navitoclax in AML cells

**Fig. 4:**
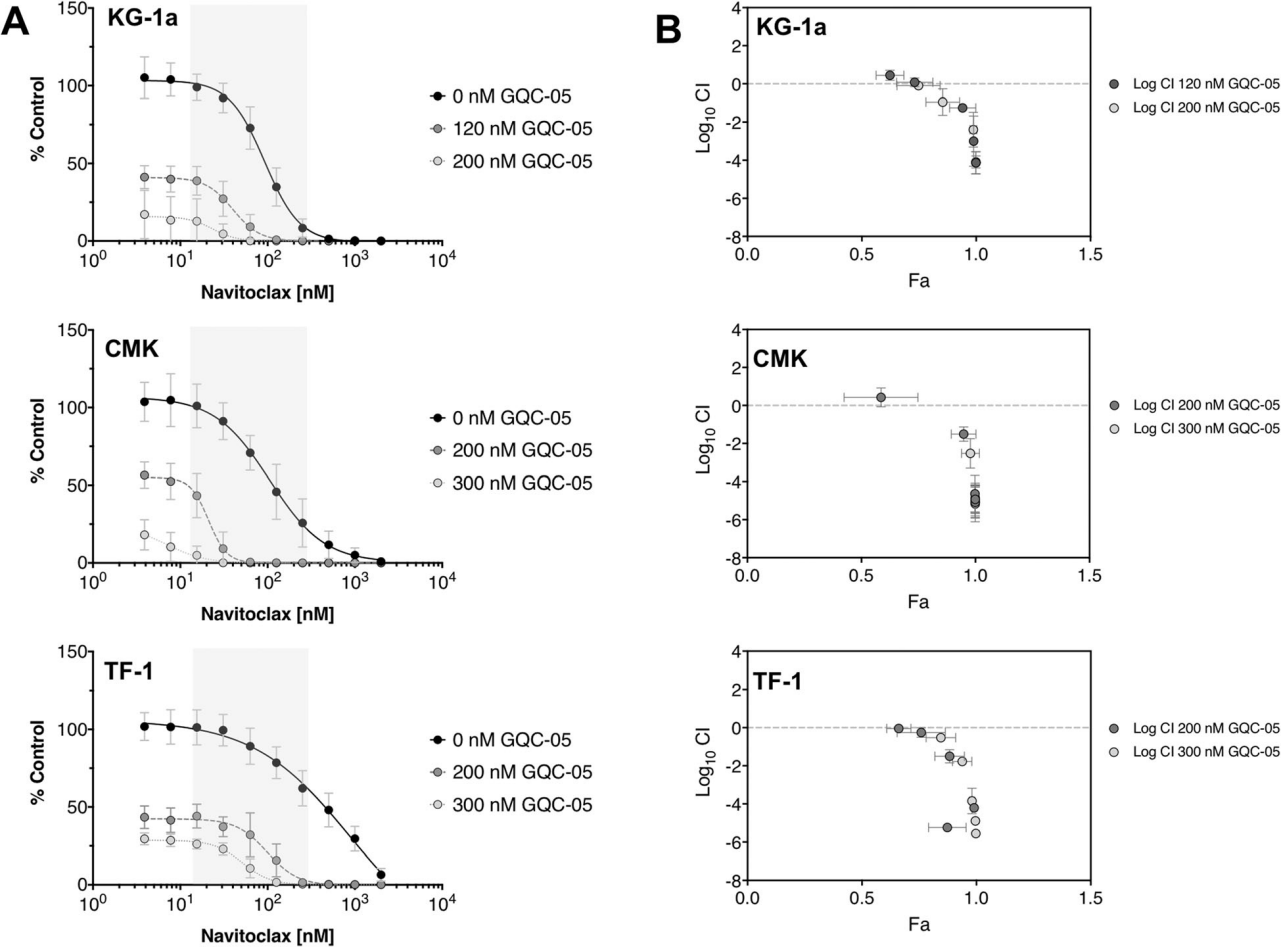
The G-quadruplex stabilizer GQC-05 is synergistic with Navitoclax in AML cell lines. The AML cell lines were treated with GQC-05 ranging from 20 to 280 nM for KG-1a and 100–600 nM for CMK and TF-1, in combination with Navitoclax ranging from 2 nM – 2 μM. Cells were assayed for cell number after 72 h of treatment and normalized to untreated cells. **a** Dose response curves for Navitoclax for each cell line in combination with no GQC-05, or approximate EC50 or EC25 doses. Doses in the shaded area were analyzed for synergy using Chou-Talalay analysis. **b** Chou-Talalay plots for each cell line treated with the combination of GQC-05 and Navitoclax where the Log_10_ of combination index (CI) is plotted against the fraction inhibited (Fa). Log_10_ CI < − 0.10 is synergistic; Log_10_ (CI) ≥ − 0.10 and < 0.08 is additive, and Log_10_ CI ≥ 0.08 is antagonistic

In order to evaluate the combination of GQC-05 with Navitoclax, drug synergy studies were performed on the AML cell lines KG-1a, CMK and TF-1. Dose response curves of Navitoclax indicated a shift in the EC50 when co-treated with GQC-05 compared to vehicle for all three AML cell lines (Fig. 4a). Synergy was assessed using Chou-Talalay analysis [17] and was seen across several combinatorial doses of GQC-05 and Navitoclax (Fig. 4b).

### The combination of GQC-05 and Navitoclax can rapidly induce apoptosis and potentiates cytotoxicity in AML cell lines

**Fig. 5:**
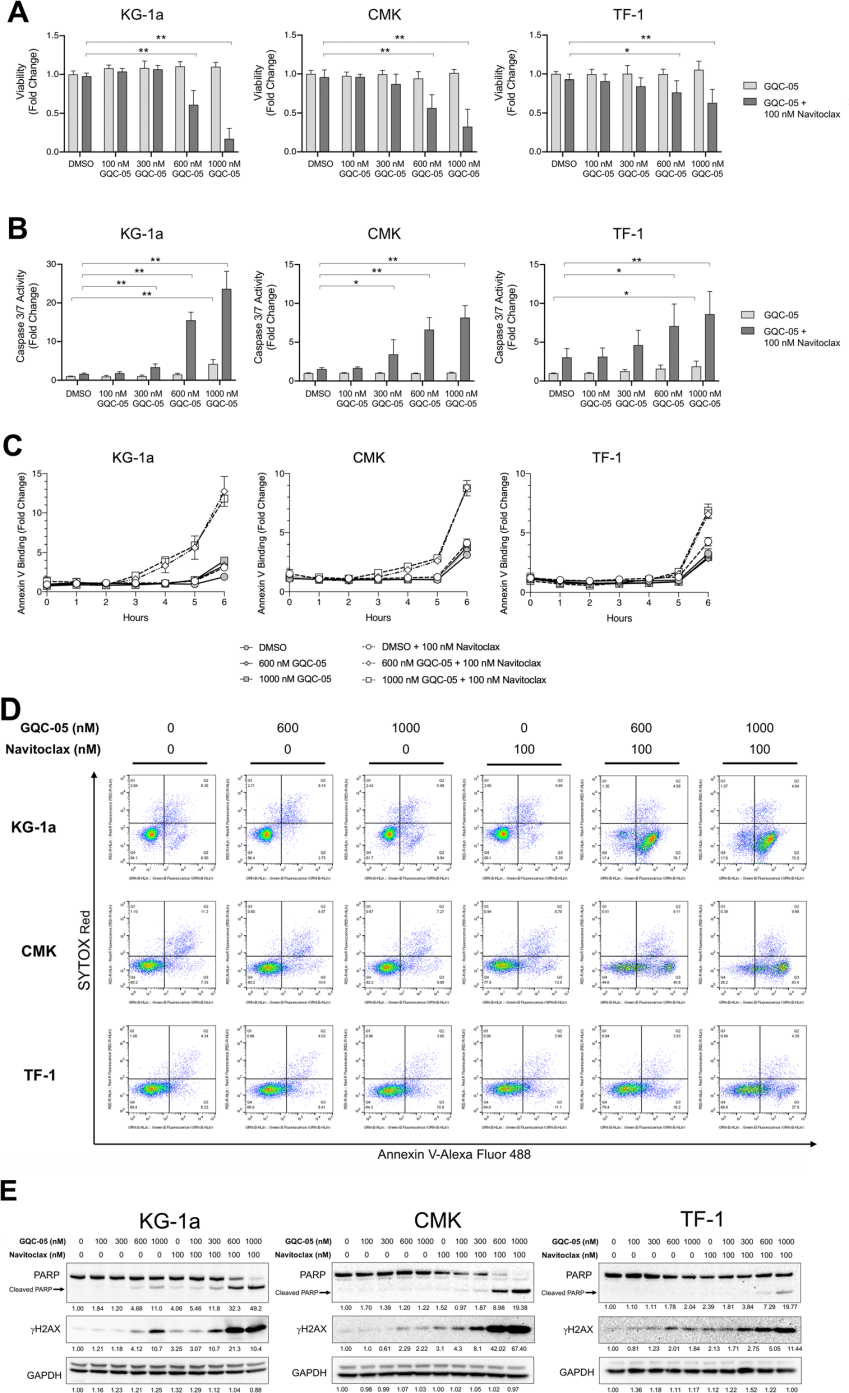
The combination of GQC-05 and Navitoclax induces rapid apoptosis, DNA damage, and potentiates cytotoxicity in AML cell lines. The AML cell lines KG-1a, CMK and TF-1 were treated with vehicle (DMSO) 100, 300, 600 and 1000 nM GQC-05 ranging in combination with either vehicle (DMSO) or 100 nM Navitoclax for 6 h. **a-b** Treated cells were assayed for (**a**) cell number and (**b**) Caspase 3/7 activity after 6 h of each treatment, normalized to vehicle treated cells and plotted as plotted as mean +/− SD. * *P* < 0.005, ** *P* < 0.0001 for quadruplicate samples of three biological replicates. **c** Kinetic analysis of Annexin V binding upon treatment of AML cells with GQC-05 and either vehicle (DMSO) or 100 nM Navitoclax. Graphs are representative of two biological replicates using triplicate wells, normalized to Time 0 and plotted as mean +/− SD. **d** Dual color flow cytometric analysis using Alexafluor 488-Annexin V and SytoxRed staining of treated cells. **e** Western immunoblotting of whole cell lysates from KG-1a, CMK, and TF-1 cells analyzed for expression of cleaved PARP and γH2AX. GADPH is shown as a loading control. Band intensities for cleaved PARP and γH2AX were calculated and normalized to GAPDH and vehicle controls

Given that the combination of GQC-05 and Navitoclax was synergistic, we analyzed the activity of the drug combination on apoptosis. Co-treatment of AML cells with GQC-05 and 100 nM Navitoclax for 6 h showed a decrease in viability at higher GQC-05 doses (Fig. 5a) and marked increase in caspase 3/7 activity (Fig. 5b) and Annexin V staining (Fig. 5c) when compared to treatment with GQC-05 alone. Kinetic studies of Annexin V binding indicated that apoptosis occurs more rapidly and at lower doses in KG-1a cells than CMK or TF-1 cells (Fig. 5d) consistent with the observation that KG-1a cells are more sensitive to GQC-05. Similarly, the combination of GQC-05 and Navitoclax for 6 h induced an increase in PARP cleavage and an increase in γH2AX in KG-1a, CMK and TF-1 cells indicating potentiation of DNA damage in the AML cell lines (Fig. 5e).

### Comparison of the combination of Navitoclax with GQC-05 to other cytotoxic chemotherapeutic agents and inhibitors

**Fig. 6:**
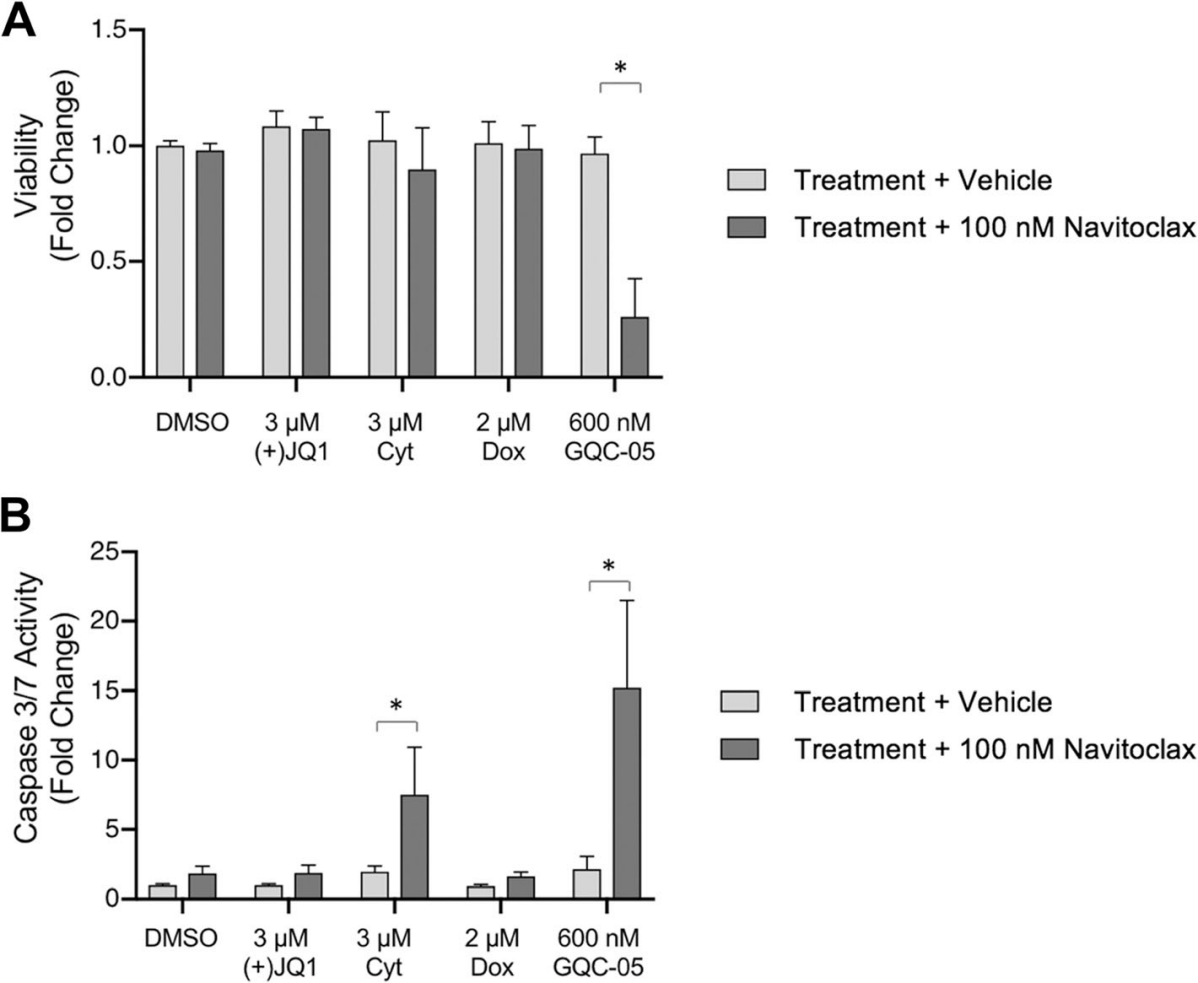
Navitoclax potentiates apoptosis and cytotoxicity greater in combination with GQC-05 than with doxorubicin, cytarabine, or (+)-JQ1. The AML cell lines KG-1a was treated with vehicle (DMSO), 3 μM (+)-JQ1, 3 μM Cytarabine (Cyt), 2 μM Doxorubicin (Dox) or 600 nM GQC-05 in combination with either vehicle (DMSO) or 100 nM Navitoclax for 6 h. **a-b** Treated cells were assayed for (**a**) cell number and (**b**) Caspase 3/7 activity after 6 h and normalized to vehicle treated cells. Quadruplicate wells were measured for each experiment and the graph of three biological replicate was plotted as mean +/− SD. * *P* < 0.0001

In order to evaluate the uniqueness in response of KG-1a cells to the combination of GQC-05 and Navitoclax, we compared the activity of the combination of Navitoclax in combination with the cytotoxic agents doxorubicin and cytarabine, as well as the BET inhibitor (+)-JQ1, a bromodomain inhibitor which is also known to downregulate *MYC* and *MYC*-dependent target genes (34). Cells were co-treated with twice the IC90 doses doxorubicin, cytarabine, (+)-JQ1, or GQC-05 combined with vehicle or 100 nM Navitoclax for 6 h. A decrease in viability was observed with the GQC-05 combination (Fig. 6a) as well as a more pronounced increase in caspase 3/7 activity (Fig. 6b) when compared to the combination of either doxorubicin, cytarabine or (+)-JQ1 and Navitoclax.

## Discussion

### Selective cytotoxicity of GQC-05 on a subset of AML cell lines

One strategy to reduce expression of oncogenes is to exploit an available small molecule that binds to and stabilizes the G4 structure on the promoter leading to the downregulation of protein expression [19]. The potential for gene specificity using this approach stems from the variable structure of G-quadruplexes. Each structure is anchored by stacked guanine tetrads, but composition and length of the loops, number of tetrads, and orientation of the strands can vary [20]. Small molecule inhibitors can be designed to preferentially bind specific structures. Given that G-quadruplexes form as a result of superhelical stress on DNA brought on by transcriptional unwinding [21] and oncogenes are transcribed at a higher rate in cancer cells, we hypothesized that there would be a higher presence of the G-quadruplex targets in cancer cells than in normal cells. This could reduce the effect of G-quadruplex targeted therapies on normal cells and potentially reduce toxicity in a clinical setting.

AML is the product of genetic alterations that cause dysregulation in the maturation of white blood cells, cell proliferation, cell survival, and apoptosis. Some of the most common mutations that result in AML are products of the following gene fusions: *FLT3-ITD* [22], *AML1-ETO*, *PML/RARa*, and *PLZF/RARa* [23]. Each underlying mutation of AML causes differential expression of many proto-oncogenes that could be used as potential targets (citation). Many AML mutations are correlated with increased expression of the proto-oncogene *MYC* making it an attractive therapeutic target in AML [12, 13, 24]. Furthermore, depletion of MYC has been shown to decrease AML cell proliferation and prolong survival of mice transplanted with AML [24]. Elevated MYC expression in AML patients is correlated with poorer prognosis.

The small molecule GQC-05 has previously been described as having G4 binding properties with a preference for the (NHE)III G-quadruplex on the *MYC* promoter sequence and demonstrated decreased *MYC* gene expression in non-Hodgkin’s lymphoma cells [11]. Given that GQC-05 induced *MYC* decreased expression and apoptosis in lymphoma cells, we investigated whether it would have similar anti-proliferative activity on AML cells.

Treatment of 14 AML cell lines with GQC-05 showed a wide range of cytotoxic activity (Table 1) and *MYC* expression. Three cell lines KG-1a, CMK and TF-1 were selected for further analysis based on their varied sensitivity to GQC-05 and expression of MYC. The promyelobalstic cell line KG-1a and the erythroblast cell line TF-1 were both derived from adult AML patients [25, 26]. The CMK cell line was derived from a megakaryoblastic pediatric leukemia patient with Down syndrome [15]. The KG-1a cell line was the most sensitive of these three lines to the cytotoxic effects of GQC-05 (Table 1). Treatment with GQC-05 showed more substantial knockdown of *MYC* mRNA and MYC protein in the KG-1a cell lines after 6-h treatment compared to CMK and TF-1 cells (Fig. 2). GQC-05 also induced apoptosis after 24 h of exposure in KG-1a cells, which was not as notable in CMK and TF-1 cells (Fig. 1a). Moreover, GQC-05 induced H2AX phosphorylation in KG-1a cells indicating that DNA damage could also be induced by GQC-05 treatment. These data suggest that reduction of MYC expression and the cytotoxic effects of GQC-05 can vary across AML cell lines and that MYC expression may not necessarily correlate to GQC-05 activity as there are likely promoters with which GQC-05 interacts in addition to MYC. Furthermore, activity of GQC-05 involves both apoptosis and DNA damage in this cellular context.

Recently, a G-quadruplex stabilizer APTO-253 was described that down regulated MYC expression and also induced DNA damage in AML cells [27], similar to what was observed by GQC-05. APTO-253, which is presently in clinical studies, was also described as inducing p21 and promoting cell cycle arrest. Although there are similarities in the activity of these two G-quadruplex binding small molecules, structurally APTO-253 is a 2-indolyl imidazole (4,5-d) phenanthroline derivative, which differs from the ellipticine GQC-05.

We sought to identify small molecules that could potentiate the activity of GQC-05, and performed combination drug screening using a cohort of inhibitors and identified the Bcl-2/Bcl-X_L_ inhibitor Navitoclax as a potentiator of GQC-05 (Fig. 3). Further analysis showed that combinations of Navitoclax and GQC-05 were synergistic (Fig. 4) and rapidly induced apoptosis and DNA damage (Fig. 5). We next aimed to determine if other agents that caused either DNA damage or decreased MYC expression could induce the rapid apoptosis and decreased cell viability when combined with Navitoclax similar to that with GQC-05. Both doxorubicin [28] and cytarabine [29] have been previously shown to induce damage DNA [28, 29], while the BET inhibitor (+)-JQ1 can down-regulate *MYC* expression [30]. Interestingly, the rapid apoptosis was predominantly seen with the GQC-05/Navitoclax combination and mildly with the cytarabine/Navitoclax combination compared to Navitoclax combinations with either doxorubicin or the BET inhibitor (+)-JQ1. The rapid decrease in cell viability was seen with only the GQC-05/Navitoclax combination, thus indicating that GQC-05 may possess additional mechanisms of cytotoxic activity. GQC-05 has previously shown to have binding activity to other G-quadruplex sites such as Bcl-2, HIF-1α, hTERT, PDGF-A, PDGF-Rβ, and potentially others [11]. The lack of cytotoxicity and apoptosis in the (+)-JQ1/Navitoclax combination further demonstrates that the synergistic effects of GQC-05/Navitoclax combination may not be MYC mediated.

Navitoclax is a potent inhibitor of both Bcl-2 and Bcl-X_L_ activity [31] and has previously been shown to be an effective partner for cytotoxic agents including cytarabine in T-ALL cells [32] and solid tumor cell lines [33]. Our data suggests that the activity of GQC-05 in combination with Bcl-2/Bcl-X_L_ inhibition promotes a more pronounced and rapid cytotoxic event in AML cells. This result is supported by a recent study using a combination of GQC-05 and a C-quadruplex (i-motif) binding small molecule that down-regulated Bcl-2 showed increased activity over GQC-05 alone in diffuse large B-cell lymphoma [34]. Another recent study showed that GQC-05 can bind to the G-quadruplex structures on the pre-mRNA of Bcl-X thus affecting RNA splicing resulting in antagonism of the anti-apoptotic Bcl-X_L_ and activation of the pro-apoptotic form Bcl-X_S_ [35]. This finding would suggest that GQC-05 could reduce the expression Bcl-X_L_ thereby lowering the threshold for Bcl-X_L_ inhibition by Navitoclax, and lead to the observed increase activity of the combined agents.

GQC-05 illustrated a marked increase in DNA damage and apoptosis in AML cell lines. Though, direct stabilization of the MYC NHEIII G-quadruplex by GQC-05 would lead to decreased MYC expression it’s also likely that GQC-05 may bind other G-quadruplex structures or DNA motifs, potentially leading to additional DNA damage.

## Conclusions

In summary, our results suggest that the G-quadruplex stabilizing compound GQC-05 has varied activity in the panel of AML cells tested. GQC-05 induced DNA damage and apoptosis, both of which are enhanced by with the addition of the Bcl-2/Bcl-X_L_ inhibitor Navitoclax. The combination effect with GQC-05 and Navitoclax is more rapid and pronounced than the combination effects of GQC-05 and other chemotherapeutic agents that are currently being used to treat AML patients. GQC-05 down regulates MYC at the mRNA and protein level, though the pronounced combinatory effects are most likely mediated through other binding sites. These additional properties would need to be further characterized to better understand the mechanism of GQC-05 and possibly similar compounds that can stabilize G-quadruplexes.

## Supplementary information

**Additional file 1: Table S1.** AUC values for Combination Drug Screen with GQC-05 in KG-1a Cells.

## Data Availability

All data generated or analyzed during this study are included in this published article and its supplementary information files.

## Abbreviations

AML: Acute Myeloid Leukemia
ATS: Acoustic Transfer System
AUC: Area Under the Curve
DMSO: Dimethyl Sulfoxide
G4: G-quadruplex
GMCSF: Granulocyte Macrophage Colony-Stimulating Factor
STR: Short Tandem Repeat
