# Supplementary material for: In vitro activity of a G-quadruplex-stabilizing small molecule that synergizes with Navitoclax to induce cytotoxicity in acute myeloid leukemia cells
Source: BMC Cancer. 2019 Dec 27;19:1251. doi: 10.1186/s12885-019-6464-9 (PMC6935221; doi:10.1186/s12885-019-6464-9)
Supplement: Supplementary file 1 — Additional file 1: Table S1. AUC values for Combination Drug Screen with GQC-05 in KG-1a Cells. [file 12885_2019_6464_MOESM1_ESM.docx]

| **Table S1: AUC values for Combination Drug Screen with GQC-05 in KG-1a Cells** | | | | | |  |  |  |
| --- | --- | --- | --- | --- | --- | --- | --- | --- |
| **Number** | **Compound** | **Inhibitor Class/Target** | **DMSO run 1** | **DMSO run 2** | **100 nM GQC run 1** | **100 nM GQC run 2** | **300 nM GQC run 1** | **300 nM GQC run 2** |
| Cmpd 01 | (+)-JQ1 | BET bromodomain | 2.800 | 2.888 | 2.819 | 2.623 | 3.311 | 3.337 |
| Cmpd 02 | 17-AAG | HSP90 | 3.991 | 4.088 | 3.956 | 3.405 | 4.491 | 3.932 |
| Cmpd 03 | Azacitidine | DNA methylation | 3.948 | 3.447 | 3.590 | 3.514 | 4.474 | 4.263 |
| Cmpd 04 | AZD4547 | FGFR | 2.102 | 2.301 | 2.451 | 2.194 | 3.249 | 3.166 |
| Cmpd 05 | AZD8055 | mTOR | 2.674 | 2.616 | 2.772 | 2.703 | 4.315 | 3.933 |
| Cmpd 06 | BGJ398 | FGFR | 1.740 | 1.572 | 1.719 | 1.809 | 2.435 | 2.457 |
| Cmpd 07 | BIIB021 | HSP90 | 2.845 | 2.655 | 2.919 | 2.643 | 2.236 | 2.967 |
| Cmpd 08 | BMS-754807 | IGF1R | 3.630 | 3.626 | 3.647 | 3.291 | 4.543 | 3.784 |
| Cmpd 09 | Bortezomib | proteosome | 0.282 | 0.277 | 0.285 | 0.293 | 0.353 | 0.352 |
| Cmpd 10 | CAL-101 | PI3K p100 | 4.153 | 3.971 | 3.772 | 4.073 | 5.045 | 4.167 |
| Cmpd 11 | Carfilzomib | proteosome | 1.991 | 2.166 | 2.178 | 1.845 | 1.953 | 1.891 |
| Cmpd 12 | Cytarabine | arabinoside | 2.925 | 2.609 | 2.788 | 2.966 | 4.132 | 3.688 |
| Cmpd 13 | Dasatinib | SRC family | 4.224 | 3.910 | 3.511 | 3.675 | 4.162 | 3.607 |
| Cmpd 14 | Daunorubicin | Topoisomerase | 2.131 | 1.939 | 1.894 | 2.015 | 2.483 | 2.267 |
| Cmpd 15 | Decitabine | DNA methylation | 2.878 | 2.513 | 2.793 | 2.917 | 3.908 | 3.404 |
| Cmpd 16 | DMSO | control | 4.056 | 4.033 | 4.031 | 4.051 | 4.029 | 4.019 |
| Cmpd 17 | Dovitinib | FLT3, cKit, FGFR | 2.781 | 2.464 | 2.483 | 2.669 | 3.255 | 2.960 |
| Cmpd 18 | Doxorubicin | Topoisomerase | 2.557 | 2.532 | 2.447 | 2.399 | 3.204 | 3.227 |
| Cmpd 19 | Erismodegib | Smo | 4.556 | 4.237 | 4.366 | 4.136 | 4.251 | 4.362 |
| Cmpd 20 | Erlotinib | EGFR | 3.930 | 3.544 | 3.868 | 3.524 | 4.080 | 4.357 |
| Cmpd 21 | Etoposide | Topoisomerase | 4.069 | 3.726 | 3.868 | 3.952 | 3.936 | 3.876 |
| Cmpd 22 | Fenretinide | retinioid | 3.537 | 3.233 | 2.951 | 3.572 | 3.421 | 3.325 |
| Cmpd 23 | Flavopiridol | CDK | 2.939 | 2.758 | 2.474 | 2.676 | 2.821 | 2.242 |
| Cmpd 25 | Lapatinib | EGFR, ErbB2 | 4.213 | 3.711 | 3.629 | 3.642 | 4.065 | 3.998 |
| Cmpd 26 | Linsitinib | IGF1R | 4.758 | 4.013 | 3.395 | 3.854 | 4.338 | 3.770 |
| Cmpd 27 | LY-294002 HCl | PI3K | 1.263 | 1.232 | 1.450 | 1.420 | 2.004 | 1.877 |
| Cmpd 28 | LY2874455 | FGFR | 4.276 | 3.744 | 3.807 | 3.790 | 4.345 | 3.684 |
| Cmpd 29 | Mithramycin A | DNA/RNA Pol | 2.353 | 2.209 | 2.175 | 2.288 | 2.452 | 2.784 |
| Cmpd 30 | MK-2206 | AKT1/2/3 | 4.195 | 3.778 | 3.748 | 4.241 | 4.595 | 4.571 |
| Cmpd 31 | Navitoclax | BCL2 | 2.955 | 2.474 | 2.378 | 2.099 | 1.607 | 1.888 |
| Cmpd 32 | Olaparib | PARP 1/2 | 4.258 | 4.188 | 4.135 | 3.721 | 4.335 | 4.061 |
| Cmpd 33 | Pacritinib | JAK2, FLT3 | 3.190 | 3.163 | 3.032 | 2.842 | 2.831 | 2.777 |
| Cmpd 34 | Palbociclib | CDK4/6 | 3.052 | 3.042 | 3.222 | 3.183 | 4.324 | 3.793 |
| Cmpd 35 | PD-173074 | FGFR | 2.400 | 2.350 | 2.573 | 2.375 | 2.934 | 2.585 |
| Cmpd 36 | PD-0325901 | MEK | 3.271 | 3.362 | 3.324 | 3.111 | 4.340 | 4.268 |
| Cmpd 37 | PF-527485 | Smo | 4.225 | 3.895 | 3.711 | 3.636 | 4.274 | 4.138 |
| Cmpd 38 | Pimasertib | MEK1/2 | 3.367 | 3.349 | 3.591 | 3.364 | 3.189 | 3.828 |
| Cmpd 39 | PLX-4720 | BRAF V600E | 3.923 | 3.990 | 3.780 | 3.459 | 4.337 | 4.160 |
| Cmpd 40 | Ponatinib | BCR-ABL | 2.092 | 2.266 | 2.323 | 2.288 | 3.283 | 3.067 |
| Cmpd 41 | Quizartinib | FLT3 | 4.216 | 4.098 | 4.145 | 4.025 | 4.481 | 4.399 |
| Cmpd 42 | SCH772984 | ERK | 3.824 | 3.515 | 3.781 | 3.638 | 3.740 | 4.401 |
| Cmpd 43 | Selumetinib | MEK1 | 3.746 | 3.699 | 3.690 | 3.717 | 4.320 | 4.407 |
| Cmpd 44 | Sorafenib | Raf-1, BRAF, VEGFR | 4.177 | 3.836 | 3.325 | 3.712 | 4.092 | 3.283 |
| Cmpd 45 | Staurosporin | PKC | 2.056 | 1.969 | 1.811 | 1.919 | 2.281 | 2.239 |
| Cmpd 46 | Temsirolimus | mTOR | 2.335 | 2.230 | 2.605 | 2.704 | 4.479 | 4.319 |
| Cmpd 47 | Topotecan | Topoisomerase | 1.189 | 1.155 | 1.232 | 1.146 | 1.642 | 1.732 |
| Cmpd 48 | Trametinib | MEK | 3.152 | 2.997 | 3.226 | 3.041 | 3.890 | 3.593 |
| Cmpd 49 | Triptolide | RNAPII | 1.334 | 1.355 | 1.467 | 1.295 | 1.717 | 1.755 |
| Cmpd 50 | U0126-EtOH | MEK1, MEK2 | 3.948 | 3.612 | 3.378 | 3.698 | 3.475 | 3.972 |
| Cmpd 51 | Vemurafenib | BRAF V600E | 4.316 | 3.853 | 3.817 | 3.766 | 3.576 | 4.608 |
| Cmpd 52 | Vincristine | microtubule | 2.561 | 2.359 | 2.746 | 2.789 | 4.183 | 3.804 |
| Cmpd 53 | Vismodegib | P-gp Hh | 4.334 | 4.183 | 4.131 | 4.271 | 4.212 | 4.244 |
| Cmpd 54 | YM155 | Survivin | 3.328 | 3.030 | 3.264 | 3.224 | 3.533 | 3.715 |
